# Applying Mathematical Tools to Accelerate Vaccine Development: Modeling *Shigella* Immune Dynamics

**DOI:** 10.1371/journal.pone.0059465

**Published:** 2013-04-02

**Authors:** Courtney L. Davis, Rezwanul Wahid, Franklin R. Toapanta, Jakub K. Simon, Marcelo B. Sztein, Doron Levy

**Affiliations:** 1 Natural Science Division, Pepperdine University, Malibu, California, United States of America; 2 Center for Vaccine Development, University of Maryland School of Medicine, Baltimore, Maryland, United States of America; 3 NanoBio Corporation, Ann Arbor, Michigan, United States of America; 4 Department of Mathematics and Center for Scientific Computation and Mathematical Modeling, University of Maryland, College Park, Maryland, United States of America; University of Melbourne, Australia

## Abstract

We establish a mathematical framework for studying immune interactions with *Shigella*, a bacteria that kills over one million people worldwide every year. The long-term goal of this novel approach is to inform *Shigella* vaccine design by elucidating which immune components and bacterial targets are crucial for establishing *Shigella* immunity. Our delay differential equation model focuses on antibody and B cell responses directed against antigens like lipopolysaccharide in *Shigella*’s outer membrane. We find that antibody-based vaccines targeting only surface antigens cannot elicit sufficient immunity for protection. Additional boosting prior to infection would require a four-orders-of-magnitude increase in antibodies to sufficiently prevent epithelial invasion. However, boosting anti-LPS B memory can confer protection, which suggests these cells may correlate with immunity. We see that IgA antibodies are slightly more effective per molecule than IgG, but more total IgA is required due to spatial functionality. An extension of the model reveals that targeting both LPS and epithelial entry proteins is a promising avenue to advance vaccine development. This paper underscores the importance of multifaceted immune targeting in creating an effective *Shigella* vaccine. It introduces mathematical models to the *Shigella* vaccine development effort and lays a foundation for joint theoretical/experimental/clinical approaches to *Shigella* vaccine design.

## Introduction

Vaccines protect millions of people from viral and bacterial infections every year [1]. Unfortunately, some diseases have defied all attempts at developing effective vaccines. Major hurdles often include highly diverse pathogen strains, imperfect experimental animal models, and a lack of specific knowledge about how the immune system fights off a given disease [2]–[4]. Mathematical modeling can help vaccine development by capturing complex immunological dynamics and highlighting which immune components are likely of greater importance in protection and disease clearance from particular pathogens. One disease for which vaccination efforts might greatly benefit from mathematical input is shigellosis, caused by *Shigella*, a bacterium that causes roughly 120 million dysentery infections and kills over 1.1 million people (predominantly young children) worldwide every year [5]. Treatment of shigellosis, which relies on the administration of antibiotics, has become increasingly difficult as resistance to both first- and second-line antibiotics has spread [4]. Thus, prevention of shigellosis is a public health priority. Unfortunately, no vaccine has been licensed against *Shigella* despite decades of clinical trials [3], and much remains unknown about how the immune system reacts to *Shigella* infections. One major hurdle in vaccine development is that identifying immunological correlates of protection in *Shigella* infections, whether mechanistic or nonmechanistic [6] has proven elusive [3].

In this paper, we create mathematical models of the immune response against *Shigella*, which we use to determine which immune effector mechanisms best confer immunity against *Shigella*. We concentrate our efforts on the humoral immune response, as current vaccines strive to incite protective immunity by eliciting specific memory B cells (B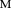) and antibody responses [3], [7]–[10].

*Shigella* infections occur via fecal-oral transmission [5]. Once ingested, *Shigella* infiltrates the gut epithelium via host M cells, which transport bacteria from the gut lumen to macrophages and other innate immune cells that reside just below the mucosal epithelial barrier in the lamina propria [4], [11]. These cells typically engulf bacteria and destroy them inside phagocytic vacuoles; however, *Shigella* is capable of escaping from these cells to invade epithelial cells from the basolateral side while also inducing macrophages to apoptose [4]. *Shigella* that evades destruction by innate immune cells in the lamina propria can enter epithelial cells, after which it can move freely inside and between epithelial cells as an intracellular pathogen [4], [11], [12]. It is believed that an innate immune response is rarely sufficient to clear a *Shigella* infection, especially after it enters epithelial cells [9]. However, as the infection progresses, *Shigella* can elicit the induction of antibody responses and effector T cells, as well as memory B and T cells, which typically results in the elimination of the organism [7]–[10], [12], [13].

Activated *Shigella*-specific B cells undergo clonal expansion, somatic hypermutation, class switching, and differentiation into antibody-secreting plasma cells (ASC) and B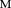 cells. Plasma cells initially produce M-type immunoglobulin (IgM); however following class switching, these cells secrete either IgG, the most abundant antibody in serum, or IgA, which is generally accepted to be the most abundant and active antibody isotype at mucosal surfaces [13]–[16]. IgA, IgG, and IgM antibodies cross the epithelial barrier to function in the lumen. This is accomplished by several mechanisms, including active mechanisms (e.g., poly-Ig receptor (pIgR) transfer for IgM and IgA and the Fc neonatal receptor (FcRn) for IgG) and passive mechanisms (paracellular pathway) for monomeric IgG and IgA [14], [15], [17]. Once in the lumen, antibodies act by coating bacteria to enhance phagocytosis and bacterial killing and/or preventing attachment to host cells. In particular, serum IgG and IgA-ASC that target *Shigella* lipopolysaccharide (LPS) have been shown to correlate with protection [3], [9], [10], [18], [19].

The role of cell-mediated immunity (CMI) in protection from *Shigella* infections is largely unknown. Elevated levels of IFN-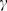 and other T-cell-derived cytokines have been proposed to play a role in control and clearance of *Shigella* infections [20]–[28]. This suggests that either cytokines control the infection directly or they are indicative of other T cell effector functions. For instance, the impact of cytotoxic T cells (CTLs), which kill infected host cells, on *Shigella* clearance is not known [4], [29].

Overall, the immune response against *Shigella* must be multifaceted to clear an infection that is both intracellular and extracellular. It is likely that in the clearance of a pathogen such as *Shigella*, antibody responses may be important in the extracellular phase while CMI may play a dominant role in the intracellular phase. An antibody-based vaccine must not only eliminate the bacteria from the gut lumen and lamina propria but should also prevent *Shigella* from entering epithelial cells. Clearance of infected epithelial cells requires a CMI response. Thus, common vaccine targets include lipopolysaccharide (LPS) on the bacterial surface as well as components of the bacteria’s type III secretion system, such as IpaB and MxiH, which are critical components of the *Shigella* machinery that allows their entry to epithelial cells [2], [3]. Having a clear understanding of which immune interactions are necessary and/or sufficient for protection could greatly aid the development of new multiple-target vaccine strategies [3].

To this end, we develop mathematical models of the immune response against *Shigella* in order to better qualitatively and quantitatively determine the effector immune responses that correlate with protection. We capture the primary immunological and bacterial dynamics with systems of ordinary and delay differential equations. Delays are employed to incorporate biological time-scale differences between existing immunity and new immune activation. Thus, in the first few days of an infection, immune protection against *Shigella* is conferred by innate immune cells or existing antibodies elicited by prior infections or vaccines; later, the presence of *Shigella* antigens elicit robust B and/or T cell responses.

While there has been much ongoing activity in deriving mathematical models of the immune interactions with other diseases (e.g., tuberculosis and influenza [30]–[35]), to the best of our knowledge, this is the first mathematical model of the immune response against *Shigella*. Furthermore, while other mathematical studies have examined the impact of vaccines on pathogen dynamics ([36], [37]), the application to *Shigella* vaccine development is novel.

We focus on *Shigella*’s interactions with the humoral immune response, which consists of antibodies (IgA and IgG, which function predominantly in the gut lumen and lamina propria, respectively), ASC, and B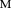 cells. We incorporate an innate immune cell compartment in which *Shigella* is subject to phagocytic killing but escapes antibody action. Notably, our model does not include cell-mediated immunity components such a CTLs that might eradicate *Shigella* bacteria inside host epithelial cells, as T cell activity against *Shigella* remains largely undefined. Using the model, we examine infection dynamics both in the presence and absence of an assumed *Shigella* vaccine directed against *Shigella* outer membrane components, such as lipopolysaccharide (LPS), that are displayed throughout an infection. We investigate whether LPS-directed antibodies are sufficient to prevent a severe *Shigella* infection. The model incorporates both mucosal (IgA) and systemic (IgG) immune responses, and we compare which is most effective. We also focus in on *Shigella*’s ability to infect host epithelial cells and ask if antibodies directed against epithelial entry are necessary components of a successful *Shigella* vaccine.

The structure of this paper is as follows: we first derive the model and explain the underlying biological dynamics that it captures. In the Analysis section, we identify the equilibria, including disease-free and vaccinated states, and evaluate their stability. In the Dynamics section, we present numerical simulations of the model to determine immune and bacterial dynamics before and after vaccination, to quantify the number of anti-LPS antibodies required for immune protection, and to explore the potential effectiveness of epithelial entry targeting. In the Parameters section, we alternately vary certain parameters to deduce their impact on model results (while leaving more rigorous sensitivity analysis to future studies) and we identify key factors that potentially correlate with *Shigella* immunity. Finally, we summarize our results and discuss their implications on the *Shigella* vaccine development process.

In the broader sense, this paper lays the foundation for determining *Shigella*’s immunological correlates of protection by developing mathematical models of the immune response against *Shigella*. Model results improve our understanding of the immune response elicited against *Shigella*, suggest alternative approaches to future vaccine design, and take the first steps in forecasting the success of their implementation.

## Methods

**Figure 1:**
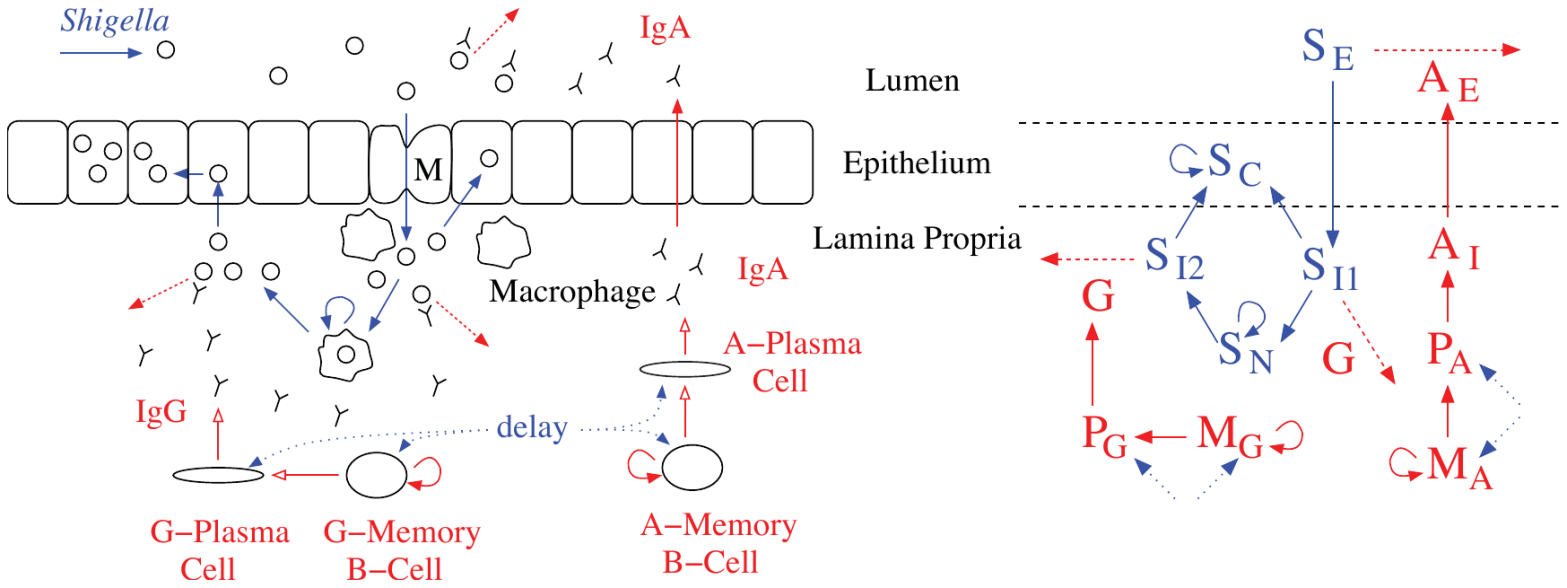
Simplified schematic of the predicted interactions of *Shigella* and the host in the gut. We translate bacterial pathogenesis (blue) plus antibody and B cell dynamics (red) seen in vivo (left) to mathematical reactions (right). The most severe symptoms result when *Shigella* escapes the humoral immune response by infecting epithelial cells. Prior to this, *Shigella* can be removed by antibodies (luminal IgA or lamina propria IgG targeting LPS in *Shigella*’s outer membrane) or engulfed by macrophages (from which it escapes or is destroyed). Delay in creation of new B cells from naive cells during infection is included in the model. Abbreviations: 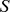: *Shigella*, 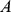: IgA, 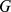: IgG, 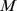: B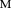, 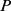: ASC, 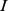: in Lamina Propria, 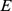: Luminal, 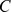: Epithelial, 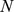 Engulfed.

We write a system of differential equations to capture bacterial and immune dynamics and focus on humoral immune components that can potentially be elicited through vaccination. Bacterial pathogenesis and the reaction diagram corresponding to the model are given in Figure 1. A primary goal of vaccination is the establishment of B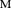 populations that maintain antibody and ASC (plasma cell) populations; thus, all three serve as model variables. Furthermore, we distinguish between IgA and IgG at both the antibody and cellular level in order to parse potential differences between mucosal (IgA) and systemic (IgG) immune responses. We do not include cytotoxic T cells, which could play a key role in controlling wild-type *Shigella* once they have invaded host epithelial cells, macrophages, and other cells, since a role for this effector CMI activity in fighting infection remains to be well characterized [3].

In our model, we incorporate delays into mathematical terms for the creation of new B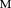 and new plasma cells from naive cells during an infection. This captures the day-or-longer time frame from naive activation to effector/memory functionality in which naive B cells proliferate and differentiate to form new cell types. We also assume a 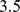-day delay from the start of a *Shigella* infection to the initialization of a new immune response; in this time window, only previously established antibodies and B cell dynamics can target the bacterial population.

Our model consists of a system of ordinary and delay differential equations that tracks bacterial populations in multiple spatial compartments as well as the immune response composed of *Shigella*-specific IgA and IgG B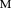, plasma cells, and antibodies. At the start of infection, *Shigella* takes advantage of a natural activity of host M cells, which shuttle material from the lumen across the epithelium to innate immune cells such as macrophages that wait in the lamina propria to engulf *Shigella* and destroy it. *Shigella* travels from the lumen, where it is denoted 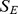, into the lamina propria (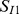) at rate 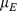, where it either is engulfed by macrophages (becoming 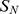) at rate 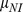 or escapes and can enter epithelial host cells (becoming 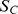) at rate 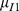. Most bacteria are typically destroyed inside macrophages; however, engulfed *Shigella* can avoid destruction and proliferate at rate 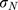 inside macrophages, where *Shigella* is safe from antibody targeting, and then escape back out into the lamina propria (becoming 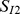) at rate 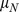. From there, we assume *Shigella* is sufficiently distant from the M cells and other macrophages to have no likelihood of re-engulfment; it instead can infect epithelial cells at rate 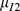 and transition to the 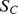 population. We will discuss later how altering this no re-engulfment assumption impacts infection dynamics.

Once within the epithelium, *Shigella* proliferates at rate 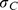 and has the ability to migrate directly between epithelial cells without reentering the lamina propria; this stage causes substantial epithelial cell destruction, which is responsible for inducing the most severe symptoms of shigellosis. To keep our first attempt at modeling *Shigella* infection relatively simple, we elected not to include in the model cell-to-cell spread or epithelial cell destruction directly (this is left to a future model) but rather assume epithelial stress correlates with significant epithelial invasion and hence high values for 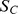. In order to prevent permanently recurring infections, we assume bacterial migration from the epithelium (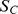) to the lumen (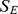) does not occur; thus, we only examine within-host dynamics, and the likelihood of person-to-person transmission along a fecal-oral route cannot be predicted with this model. By similar reasoning, we do not include the small likelihood of luminal *Shigella* proliferating before entering the lamina propria in order to avoid mathematically establishing a luminal *Shigella* population that persistently spawns new infections.

The equations governing the bacterial dynamics are as follows:
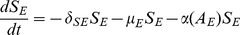
(1)

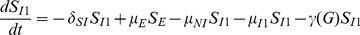
(2)

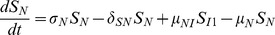
(3)

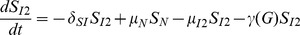
(4)

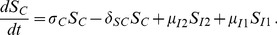
(5)


Here, the death rates (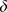 terms) incorporate *Shigella* death due to macrophage activity or other causes; *Shigella* removal via antibody is modeled separately. 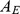 and 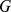 are luminal IgA and lamina propria IgG, respectively, and their dynamics are governed by the immune response equations below. We assume these antibodies recognize and bind specifically to *Shigella* outer membrane components such as LPS, which is constantly displayed and available for antibody targeting whenever *Shigella* resides in the lamina propria or lumen. When sufficient numbers (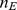 or 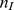, respectively) of IgA or IgG bind to a *Shigella* bacterium, the bacterium is removed or destroyed. We assume the null model of linear interaction terms as the functional forms capturing bacterial removal via antibodies; that is, 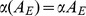 and 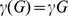. These terms could become more complex in future work if that is needed to better match with experimental or clinical dynamics.

The humoral immune response to *Shigella* consists of IgG-type B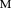 (denoted 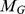), IgG-secreting plasma cells (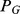), and the IgG antibodies themselves (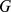), in the lamina propria. IgA antibodies are formed in the lamina propria from IgA-secreting plasma cells (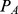), which are often derived from IgA-type B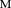 cells (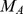); IgA (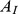) then reaches the lumen by crossing the epithelial barrier (via interactions with the pIgR receptors or the paracellular pathway, becoming 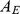), where IgA functions primarily. Antibody secretion by each plasma cell occurs constantly at rate 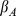 or 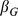, regardless of infection status, just as memory cells differentiate to form 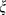 plasma cells at a low rate 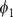 even without an infection currently occurring. However, the presence of bacteria within the lamina propria stimulates further B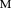 differentiation into plasma cells (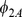 and 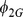 functions) while also stimulating naive B cells to create new plasma cells (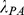 and 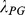 functions) and new B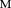 cells (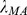 and 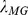 functions). In the model, the latter four terms incorporate a time delay, 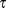, to allow time for the window between naive-cell activation and differentiated-cell functionality, as described earlier. In the absence of an infection, B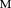 cell generation, death, and differentiation rates must balance to establish a nontrivial (nonzero) memory population that neither expand nor shrink over time. To prevent the system from becoming neutrally stable when this occurs, we assume B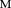 follow logistic growth dynamics.

Summarizing these immune dynamics are the following model equations:
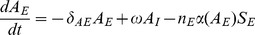
(6)

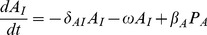
(7)

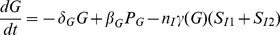
(8)

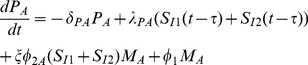
(9)

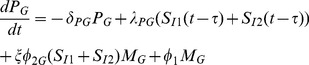
(10)

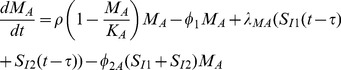
(11)

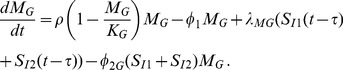
(12)


Here the functional forms are again assumed to be linear. We analyze the coupled bacterial and immune equations both analytically and numerically to discern the behavior of the model under different disease conditions. Whenever possible, parameter values have been chosen *a priori* from empirically realistic values found in the literature; they are summarized in Table 1. No parameters have been fitted.

**Table 1:** Parameter Values Used In Simulations.

Param	Value Used	Description	Reference or Note
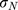	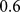 /d	*Shigella* reproduction rate in innate immune cells	high but brief [44]
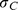	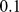 /d	*Shigella* reproduction rate in epithelial cells	high [45], [46] (smaller for
			numerical stability w/o CTLs)
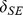	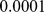 /d	Natural death rate of *Shigella* in lumen	minimal
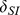	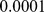 /d	Natural death rate of *Shigella* in lamina propria	minimal
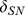	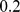 /d	Death rate of *Shigella* in macrophages	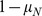
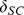	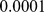 /d	Natural death rate of *Shigella* in epithelium	minimal
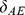	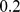 /d	Natural decay/removal rate of IgA in lumen	3.5 d hl (from 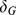 )
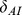	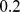 /d	Natural decay/removal rate of IgA in lamina propria	3.5 d hl (from 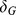 )
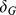	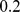 /d	Natural decay/removal rate of IgG in lamina propria	3.5 d hl (18 d hl [42]-localiz.)
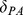	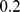 /d	Natural death rate of IgA-plasma cells	3 d hl (early pc) [42], [47], [48]
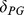	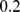 /d	Natural death rate of IgG-plasma cells	3 d hl (early pc) [42], [47], [48]
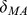	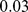 /d	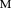 Natural death rate of IgA-B	3 wk hl [49]
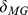	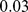 /d	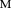 Natural death rate of IgG-B	3 wk hl [49]
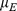	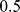 /d	Migration rate of *Shigella* from lumen to lamina propria	low ( 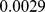 % of 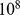 bact/mL)
			in first 2 h [50]
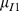	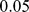 /d	Migration rate of never-engulfed *Shigella* from	
		lamina propria to epithelium	
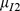	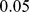 /d	Migration rate of once-engulfed *Shigella* from	
		lamina propria to epithelium	
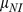	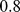 /d	Rate *Shigella* in lamina propria are engulfed by macrophages	fast
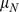	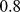 /d	Rate *Shigella* escape out of macrophages into lamina propria	fast (macs apoptose in 8 h) [44]
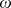	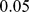 /d	Migration rate of IgA from lamina propria to lumen	20% of total IgA to lumen
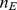	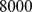 Ab/bact	Number of IgA that neutralize a luminal bacterium	(from 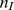 )
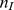	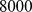 Ab/bact	Number of IgG that neutralize a lamina propria bacterium	[51]
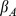	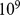 /d	Production rate of IgA by IgA-plasma cells	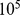 /sec [42], [52]–[55]
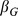	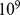 /d	Production rate of IgG by IgG-plasma cells	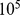 /sec [42], [52]–[55]
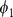	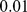 pc/mc/d	Antigen-independent differentiation rate of	
		B 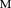 into plasma cells	
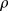	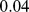 /d	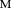 Antigen-independent cycling rate of B	
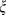	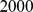 pc/mc	Number of plasma cells generated by proliferating	
		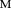 antigen-activated B	
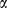	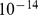 /Ab/d	Rate antibodies neutralize *Shigella* in lumen	
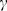	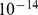 /Ab/d	Rate antibodies neutralize *Shigella* in lamina propria	
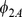	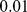 /bact/d	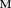 Antigen-dependent rate function for IgA-B	
		differentiation into plasma cells	
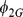	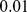 /bact/d	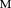 Antigen-dependent rate function for IgG-B	
		differentiation into plasma cells	
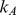	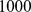 mc	IgA-B 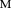 carrying capacity	
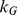	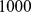 mc	IgG-B 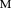 carrying capacity	
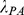	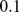 pc/bact/d	Creation rate of new IgA-plasma cells from naive B cells	
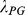	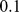 pc/bact/d	Creation rate of new IgG-plasma cells from naive B cells	
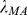	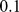 pc/bact/d	Creation rate of new IgA-B 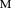 from naive B cells	
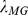	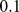 pc/bact/d	Creation rate of new IgG-B 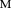 from naive B cells	
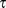	1 d	Time delay for new cell creation from naive B cells	
–	3.5 d	Initial delay until naive B cell activation in an infection	
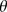	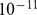	Nonmechanstic term governing the effect of	
		IgG targeted at epithelial entry	
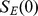	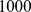 bact	Initial number of luminal *Shigella*	1000 minimum [19], [38]–[40]

Parameter values used in the model are given along with their descriptions. Applicable references or notes are given. Uncited parameters were chosen *a priori* and are not fitted. The initial condition for establishing a (luminal) *Shigella* infection is given. Other initial conditions are taken from the equilibria in Table 2. Abbreviations: d: day, Ab: antibodies, pc: plasma cells, mc: B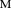 cells, bact: *Shigella* bacterium, hl: half-life.

## Results

### Analysis

Equilibrium analysis elucidates model dynamics between infections. Prior to a primary *Shigella* infection, all variables lie at a trivial equilibrium where no *Shigella* bacteria or immune components specific for *Shigella* yet exist. (The trivial equilibrium disallows potential cross-reactivity with pre-existing antibodies created in response to a different bacterial infection. Inclusion of these must be done externally to the model via initial conditions.) A *Shigella* infection is cleared unless the disease is so acutely severe that it kills the host; however, a B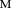 population specific for the bacteria remains indefinitely and supports ongoing plasma cell and antibody creation. The model captures this behavior with its disease-free equilibria that have nonzero numbers for antibodies, plasma cells, and B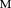 while simultaneously having no *Shigella* bacteria in any spatial compartment. Three disease-free equilibria exist for the model: one with mucosal (IgA-type) but not systemic (IgG-type) immunity, one with systemic but not mucosal immunity, and a joint equilibrium with both. The values of the model variables at equilibrium are given in Table 2; these are evaluated at the parameter values in Table 1.

**Table 2:** Equilibrium Values.

	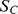 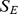 , 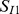 , 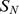 , 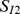 ,	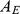	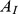	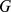	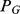 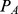 ,	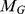 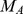 ,
Trivial						
(Pre-Infection)	0 Bacteria	0 Ab	0 Ab	0 Ab	0 Cells	0 Cells
Disease-Free:						
IgA only	0 Bacteria	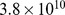 Ab	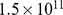 Ab	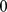 Ab	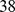 Cells,	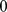 Cells
(Post-Infection)						
Disease-Free:						
IgG only	0 Bacteria	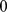 Ab	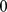 Ab	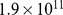 Ab	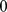 Cells, 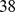 Cells	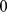 Cells, 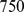 Cells
(Post-Infection)						
Disease-Free:						
IgA and IgG	0 Bacteria	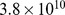 Ab	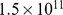 Ab	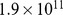 Ab	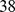 Cells	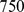 Cells
(Post-Infection)						
Nontrivial						
(Chronic Infection)			none			
	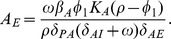					
Nonzero Terms:	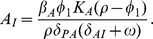		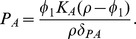		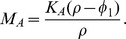	
	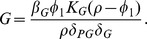		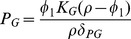		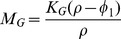	

Values of model variables at the equilibria are given. The equilibria are evaluated at the parameters in Table 1. Abbreviation: Ab: antibodies, 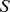: *Shigella*, 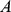: IgA, 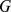: IgG, 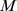: B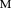, 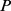: ASC, 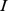: in Lamina Propria, 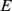: Luminal, 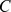: Epithelial, 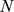 Engulfed.

Importantly, there is no completely nontrivial equilibrium of the model, which is consistent with the fact that a *Shigella* infection is never persistent nor chronically latent. If, however, macrophage re-engulfment of *Shigella* in the lamina propria is allowed–that is, if 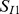 and 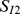 are combined into 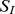 with 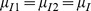
–then a persistently infected macrophage population develops that continually seeds the *Shigella* infection and prevents bacterial clearance (not shown). This chronic state is not observed biologically; hence, macrophage re-engulfment is not allowed in the model and the *Shigella* populations in the lamina propria are separate.

Stability of the trivial and disease-free equilibria can be determined by examining the eigenvalues of the Jacobian matrix for our system of differential equations at the equilibria. Since there is no truly nontrivial equilibrium and hence the *Shigella* populations are zero at all equilibria, we examine the stability of the reduced system with 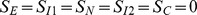. For a generic equilibrium with B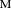 numbers 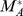 and 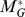, the Jacobian matrix for this disease-free system is
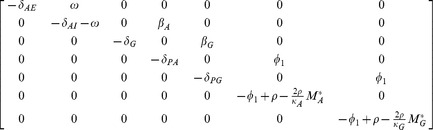
and the eigenvalues of the reduced system are



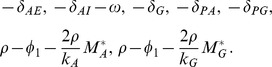



If the following two stability conditions are satisfied, the eigenvalues are all negative and hence the corresponding equilibrium is stable:
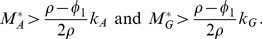
(13)


For our parameters, this means that stability occurs exactly when both 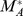 and 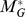 exceed 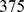 cells. Therefore, from the parameterized equilibrium values in Table 2, it is apparent that the joint (IgA and IgG) disease-free equilibrium is stable while the trivial equilibrium, IgA-only disease-free quilibrium, and IgG-only disease-free equilibrium are saddle points.

Thus, following a primary *Shigella* infection, the trivial equilibrium is not maintained and some level of permanent immunity is established. If only A-type or G-type immunity is generated initially, another infection could boost the system to the stable joint disease-free immune state. Whether these immune levels are sufficient to confer protective immunity to the host remains to be determined. Nevertheless, the model supports the hypothesis that a vaccine, which perturbs the immune system away from the trivial equilibrium, will establish a persistent nontrivial level of humoral immunity specific for *Shigella*.

### Dynamics

We assess the behavior of the mathematical model via numerical simulations. Using a delay differential equation solver in MATLAB, we examine the bacterial and immune dynamics during a *Shigella* infection that occurs either prior to or months after the administration of a vaccine; the action of the vaccine mathematically is to shift the system from the trivial equilibrium to a disease-free equilibrium. Therefore, a primary *Shigella* infection initializes with trivial variable values while a post-vaccine *Shigella* infection starts at a disease-free equilibrium. (We investigate a vaccine that elicits both IgA and IgG, and accordingly the model initializes at the joint IgA-IgG disease-free equilibrium for a post-vaccine infection.) Parameter values are given in Table 1, and their impact is discussed in the Parameters section.

**Figure 2:**
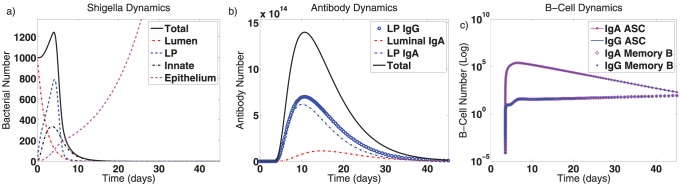
Dynamics of the mathematical model for a primary infection. Here, the model is initialized at a fully naive state (the trivial equilibrium) with 1,000 luminal *Shigella* bacteria. Resulting model dynamics over 45 days are displayed. (a) Bacterial dynamics are shown in the lumen, in the lamina propria (LP, which combines population numbers before and after macrophage engulfment), inside innate immune cells (macrophages) during engulfment, and in epithelial cells. (b) Antibody dynamics are shown for lamina propria IgG, lamina propria IgA, and luminal IgA. Total antibody levels are also given. (c) ASC and B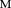 dynamics are given on a log scale. These are separated into IgA- versus IgG-type cells. The populations equilibrate to the values shown in Table 2.

We establish an infection by assuming enough *Shigella* is ingested to introduce a population of 1,000 bacteria into the gut lumen. Since a minimum of 100–1000 *Shigella* bacteria clinically cause disease, this is sufficient to cause infection [19], [38]–[40]. When invading bacteria meet a naive immune system, the dynamics in Figure 2 result. *Shigella* grows and migrates unfettered in the host during the incubation period before sufficient numbers of naive B cells have been stimulated to create ASC and B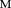 cells that target the infection. We have built a 3.5-day incubation period into the model prior to immune initiation; after this time, a B cell and antibody response is generated that eliminates the bacterial infection. IgA and IgG levels peak roughly 10–21 days after infection and equilibrate after about one month. Plasma cells and B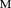 cells also reach new homeostatic levels after about a month. Crucially, these levels are consistent with a disease-free equilibrium’s nontrivial immune levels rather than with the trivial equilibrium at which the system began. Immune activity clears the *Shigella* infection to below one bacterium in the lumen and lamina propria in 20 days, but bacteria that enter epithelial cells escape humoral immune targeting and grow without restraint. It should be noted that *Shigella*’s infection of the epithelium may be controlled by cytotoxic T cells and thus in reality this epithelial population would be more controlled; however, T cell activity against *Shigella* remains largely undefined; thus we have not included them in the model. As a result, true *Shigella* dynamics in response to both humoral and cell-mediated immune responses are outside the purview of this model. For our purposes, the essential fact shown by the primary infection model is that *Shigella* can survive well in the epithelium and thus a vaccine that effectively protects against shigellosis must prevent most epithelial entry.

**Figure 3:**
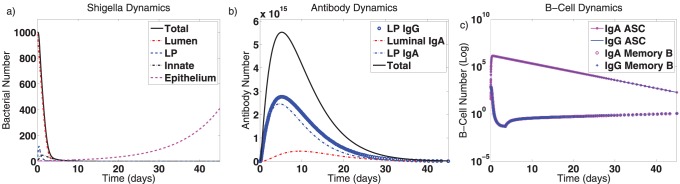
Dynamics of the mathematical model for a secondary or post-vaccine *Shigella* infection. Mathematically, a vaccine (which targets *Shigella* outer membrane components such as LPS) shifts the system to its nontrivial immune equilibrium. Here, the model is initialized at the joint IgA-IgG disease-free equilibrium with 1,000 luminal *Shigella* bacteria. The duration and severity of infection decrease and the immune response is boosted in comparison with a primary infection (Figure 2). Abbreviations: LP: lamina propria, ASC: antibody-secreting cells.

Secondary immune responses are elicited when a vaccine is given that elicits LPS-directed IgA and IgG humoral immune responses and shifts the immune system to the joint disease-free equilibrium. After the host system re-equilibrates, 1,000 wild-type *Shigella* bacteria are introduced to the gut. The dynamics of this secondary *Shigella* infection are depicted in Figure 3. A larger absolute immune response, as evidenced by the antibody and B cell peaks, is stimulated by this secondary infection (Figure 3b); this is consistent with the fact that memory cells elicit stronger and more rapid reactions than naive cells. The vaccine decreases the duration of the bacterial infection by nearly half–from 20 days to 11.5 days until complete clearance (Figure 3a). It also lessens the severity of the infection, as seen by comparing the height of the “lamina propria” and “innate” *Shigella* peaks. (The luminal peak is fixed to 1,000 by initial conditions.) Also noteworthy are the slowed dynamics of the epithelial cell infection, which suggests the host may experience temporarily reduced symptoms resulting from epithelial destruction. Nevertheless, the vaccine does not fully prevent the epithelial infection, which means the host must instead hope that a primary cytotoxic T cell response can clear it.

**Figure 4:**
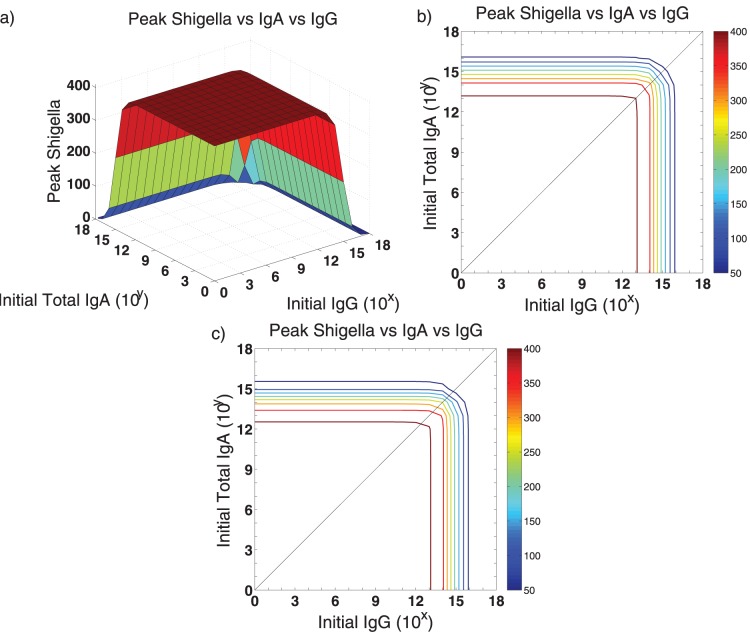
The number of antibodies needed to control an epithelial *Shigella* infection is shown. We examine the quantity of IgA and/or IgG antibodies that must be present prior to a post-vaccine or secondary infection to sufficiently contain the infection. We initialize the model with IgG and/or total IgA numbers each varying from 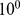 through 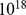 antibodies and display the peak number of non-luminal *Shigella* organisms by day 45. The unboosted model’s post-vaccine equilibrium of 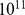 antibodies is four orders-of-magnitude less than what is necessary to keep the *Shigella* epithelial population below 100 bacteria. (a,b) Figure b displays horizonal slices through the surface in a. With the parameters in Table 1, 20% of total IgA reaches the lumen, where it functions; thus, we fix the ratio of initial luminal IgA to total IgA at 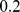. Comparing x- and y-intercepts for each contour line reveals that with only 20% of IgA functional, slightly more total IgA alone is needed to be as effective as IgG alone. (c) We change the ratio of initial luminal IgA to total IgA to 1, and thus 100% of total IgA reaches the lumen and is functional. The surface that was sliced to create c is not shown. The resulting intercepts for peak day-45 *Shigella* numbers reveal that with the number of functional IgA identical to the number of functional IgG, IgA is slightly more effective than IgG.

We next ask if *Shigella*’s infection of epithelial cells could be prevented purely by anti-LPS antibodies if they were available in larger supply (Figure 4). Here, we boost antibody numbers above vaccine levels, for instance via a pre-infection serum injection of antibodies, while B cell numbers remain at levels established by a vaccine. We examine how many IgA and IgG molecules must be present to restrain *Shigella*’s epithelial population to a given day-45 threshold. By day 45, a wild-type *Shigella* infection will have cleared; thus, we assume that if we can control the bacterial population for long enough through antibody responses, other host’s immune responses such as CMI (e.g., cytotoxic T cells or production of IFN-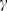 and other pro-inflammatory cytokines that activate macrophages and enhance their ability to kill intracellular *Shigella*) will be sufficient to clear the infection. In fact, recent publications have begun to show that CMI responses are important. For example, the clearance of a primary *Shigella* infection is impaired in the absence of T cells. Additional studies demonstrated that following reinfection, IL-17A and IL-22 producing T cells are primed by *Shigella* and the IL-17A produced by these cells restricts bacterial growth [41]. Furthermore, elevated levels of IFN-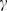 have been shown in humans with shigellosis or following administration of candidate attenuated *Shigella* vaccines [20]–[27].

The maximum tolerable day-45 threshold should be fixed as a number of *Shigella* that can infect the epithelium without the host becoming severely symptomatic; as this value is unknown, we vary the peak number of bacteria allowed and examine the corresponding antibody requirements (Figure 4). From Table 2, we know that in the order of 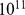 IgA and IgG established via vaccine are sustained at the joint disease-free equilibrium. Yet from Figure 4, it is clear that holding *Shigella* to small numbers requires much higher initial antibody levels; for instance, it takes 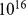 IgA alone, 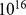 IgG alone, or 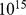 IgA and IgG together in the GI tract to keep the *Shigella* epithelial population at day 45 below 100 bacteria. This four-orders-of-magnitude increase in initial antibody levels could be difficult to elicit biologically and may be untenable.

From Figure 4 we can also parse the relative effectiveness of IgA versus IgG. We assume equal rate parameters but different spatial dynamics for IgA and IgG; IgG removes bacteria in the lamina propria while IgA is made in the lamina propria but functions in the lumen. Figure 4b displays horizontal slices through the surface in Figure 4a. The center diagonal shows where equal amounts occur. From Figures 4a and 4b, we see that IgA and IgG are nearly equally effective alone, with IgG being slightly more potent. However, we must examine the details more carefully to discern true differences in antibody efficacy. These figures show the total amount of IgA in comparison with IgG. However, IgA is distributed across two spatial compartments: the lamina propria, where it is formed, and the lumen, where it functions antimicrobially. The amount of IgA in the lumen versus the lamina propria initially is consistent with the ratio from Figure 2, in which 20% of total IgA is present in the lumen at homeostasis. Thus, in Figures 4a and 4b, 20% of the total IgA acts nearly comparably to 100% of IgG, which does not have spatial compartmentalization. This suggests that A-type antibodies may actually be more effective than G-type on a per-molecule basis. We check this by repeating the simulation done to create Figures 4a and 4b but instead requiring that 100% of the total IgA migrates to the lumen. Figure 4c shows that this has little overall effect, but careful examination of the intercepts shows that IgA is now slightly more potent than IgG. Thus, if only one type of antibody response can be elicited by a vaccine, either a mucosal (IgA) or a systemic (IgG) response will be about equally effective. An IgA-only response may require more total antibodies to sustain a sufficient luminal concentration, but each individual IgA molecule is indicated to be slightly more efficacious. If both IgA and IgG can be elicited instead of only one, there is an order-of-magnitude drop in the total amount of antibody needed for protection.

Another option to improve the efficacy of the vaccine’s control on the epithelial invasion is to modify the vaccine targets. We have shown that large amounts of IgA and IgG must be present for a vaccine targeting *Shigella* outer membrane components such as LPS to be effective. What if we additionally include an antibody population capable of specifically targeting epithelial entry? To examine this question, we alter the model to allow IgG to nonmechanistically modulate the rate at which in the lamina propria *Shigella* enters epithelial cells. Equation 5 becomes
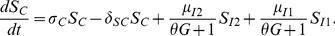
(14)


**Figure 5:**
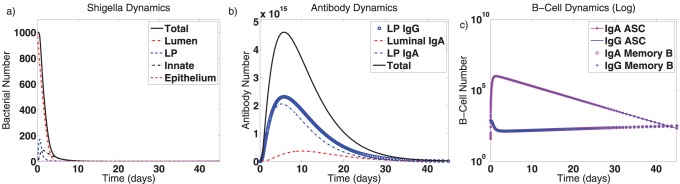
Bacterial and immune dynamics when the model includes antibody targeting of epithelial entry. If the model allows for IgG to nonmechanistically modulate the rate at which lamina propria (LP) *Shigella* enters epithelial cells, these post-vaccine dynamics result. Notably, the epithelial bacterial population is restricted to nearly zero levels.

The inclusion of epithelial targeting by antibodies has the desired effect of almost entirely preventing bacterial invasion of epithelial cells, as can be seen in Figure 5. Although the epithelial population is fractionally higher than zero, this negligible bacterial population that succeeds in circumventing these tightened immune constraints will likely be eliminated through other host defenses. It should be noted that in this altered model we imperfectly assume that the same IgG population can target both LPS and epithelial entry; a future, mechanistic model will separate these populations. However, this simple, nonmechanistic approach demonstrates that targeting epithelial entry can be a successful strategy in theory and is worth pursuing in more detail. Future work must also take into account other important issues, such as potentially brief availability of epithelial entry proteins.

### Parameters

The model parameters have been chosen from the literature whenever possible (Table 1). No parameters have been fitted. While a detailed search of parameter space is outside the scope of this current study, we explore the role of individual model parameters on the model dynamics by conducting further simulations in which we vary a single parameter while leaving the rest at the values in Table 1. We monitor the post-vaccine dynamics over 45 days. For any chosen parameter range, we measure and plot.

1. The magnitude and timing of peak total antibody numbers.
2. The magnitude and timing of the peaks in *Shigella* numbers in each spatial compartment.
3. The time of extinction of *Shigella* in non-epithelial compartments, which we define as having less than one *Shigella* bacterium total in the lumen, lamina propria, or engulfed populations.
4. The time until antibody decays to 10% of its peak value.

**Figure 6:**
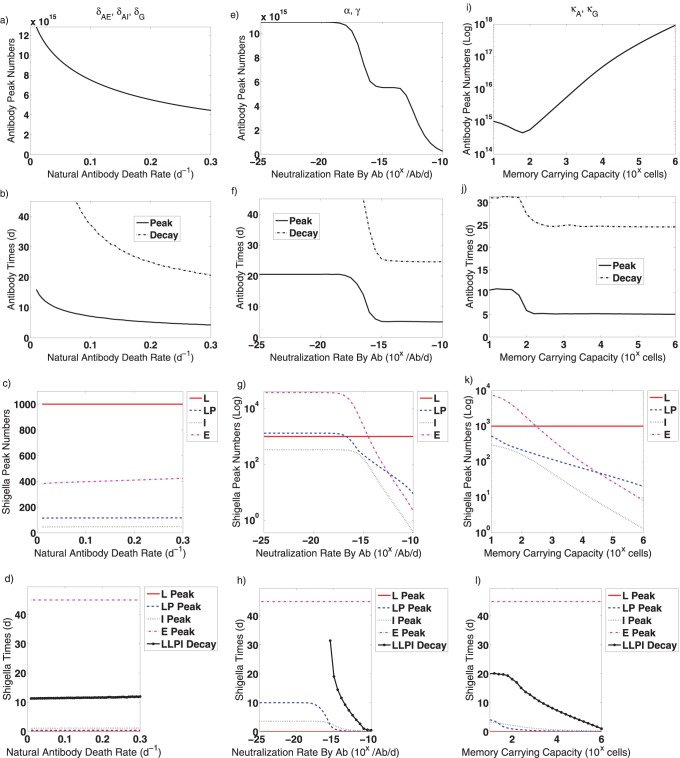
Effects of various parameters on post-vaccine dynamics are evaluated. Only one parameter type varies in each figure; other parameters are fixed to the values given in Table 1. (a–d) The natural antibody death rates (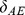, 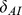, and 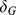) are varied together from 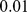 to 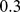/d. (e–f) The rates that a *Shigella* bacterium is neutralized by antibody (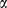 for IgA and 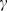 for IgG) are varied together from 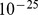 to 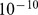/Ab/d. (i–l) The B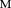 carrying capacities (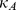 and 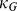) are varied from 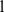 to 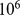 cells. The following results are tracked for each parameter: (a,e,f) the peak number of total antibody (lamina propria IgG plus lamina propria and luminal IgA), (b,f,j) the timing of the antibody peak, (c,g,k) the peak number of *Shigella* in the lumen (L), in the lamina propria (LP), engulfed in innate immune cells (I), and in the epithelium (E), (d,h,l) the timing of the *Shigella* peak in the aforementioned compartments as well as the time at which the total number of non-epithelial *Shigella* drops below one bacterium (LLPI Decay). Abbreviations: Ab: antibodies, d: day, L: lumen, LP: lamina propria, I: engulfed in innate immune cells, E: epithelium, Log: logarithmic scale, 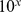: the number on the x-axis should be used as the exponent of 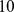 to obtain the true value.

**Figure 7:**
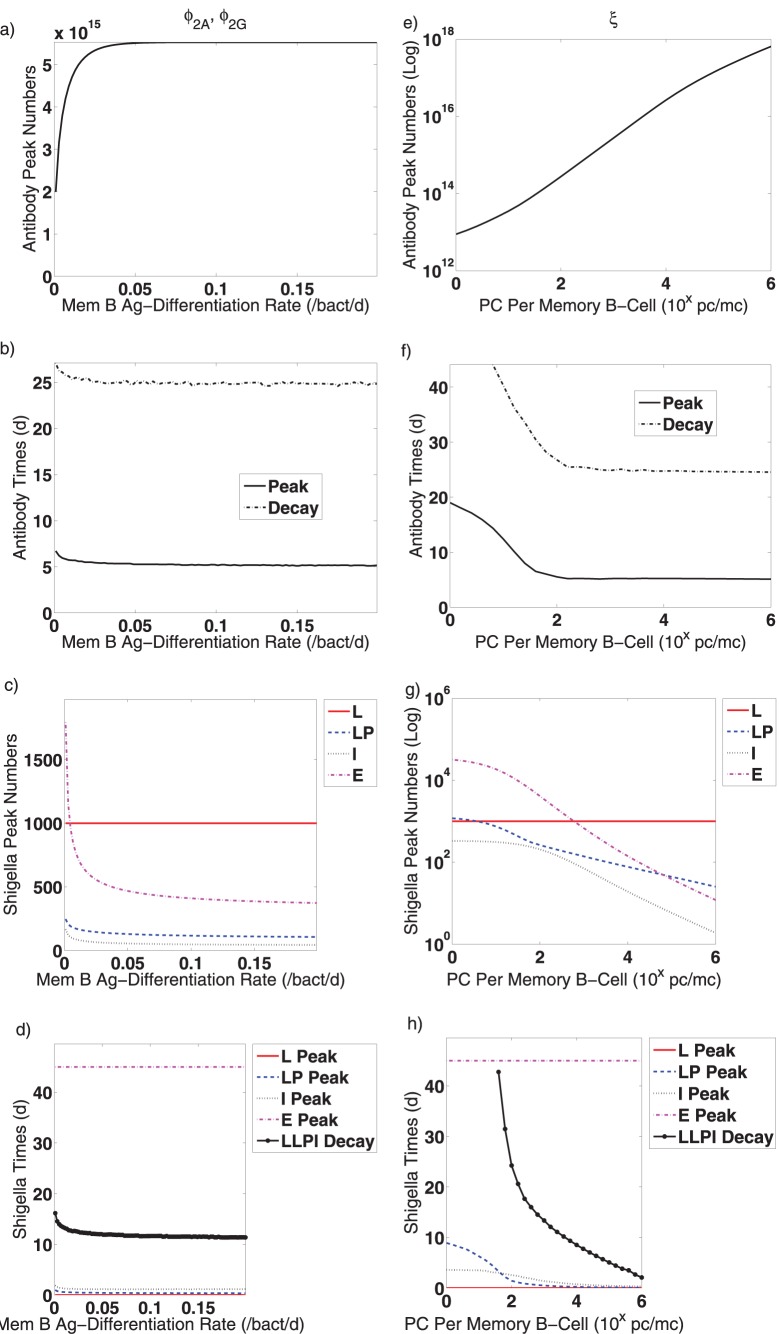
Effects of various parameters on post-vaccine dynamics are evaluated. Only one parameter type varies in each figure; other parameters are fixed to the values given in Table 1. (a–d) The rates that B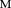 differentiate into plasma cells in the presence of antigen (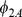 and 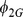) are varied together from 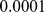 to 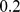/bact/d. (e–f) The number of plasma cells generated by proliferating antigen-activated B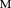 (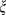) is varied from 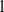 to 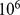 pc/mc. The following results are tracked for each parameter: (a,e,f) the peak number of total antibody (lamina propria IgG plus lamina propria and luminal IgA), (b,f,j) the timing of the antibody peak, (c,g,k) the peak number of *Shigella* in the lumen (L), in the lamina propria (LP), engulfed in innate immune cells (I), and in the epithelium (E), (d,h,l) the timing of the *Shigella* peak in the aforementioned compartments as well as the time at which the total number of non-epithelial *Shigella* drops below one bacterium (LLPI Decay). Abbreviations: Ab: antibodies, d: day, bact: bacteria, pc: plasma cells, mc: memory B cells, L: lumen, LP: lamina propria, I: engulfed in innate immune cells, E: epithelium, Log: logarithmic scale, 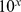: the number on the x-axis should be used as the exponent of 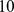 to obtain the true value.

**Figure 8:**
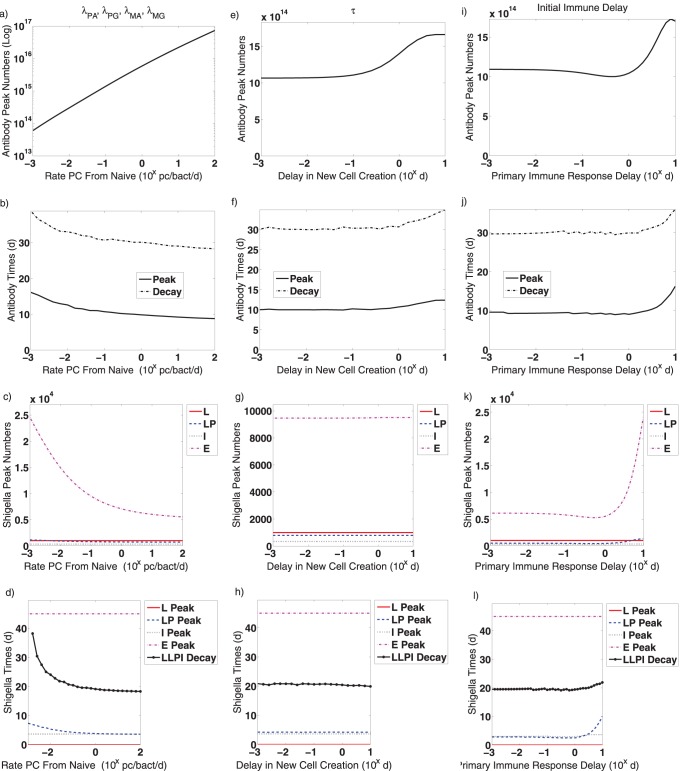
Effects of various parameters on primary infection dynamics are evaluated. Only one parameter type varies in each figure; other parameters are fixed to the values given in Table 1. (a–d) Creation rates of new plasma cells from naive B cells during an infection (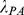, 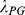, 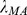, and 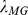) are varied together from 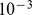 to 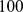 pc/bact/d. (e–f) The time delay for new plasma or B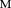 creation from naive B cells (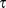) is varied from 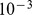 to 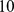 d. (i–l) The initial delay until naive B cell activation in an infection is varied from 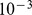 to  d. The following results are tracked for each parameter: (a,e,f) the peak number of total antibody (IgG plus IgA), (b,f,j) the timing of the antibody peak, (c,g,k) the peak number of *Shigella* in the lumen (L), in the lamina propria (LP), engulfed in innate immune cells (I), and in the epithelium (E), (d,h,l) the timing of the *Shigella* peak in the aforementioned compartments as well as the time at which the total number of non-epithelial *Shigella* drops below one bacterium (LLPI Decay). Abbreviations: Ab: antibodies, d: day, bact: bacteria, pc: plasma cells, L: lumen, LP: lamina propria, I: engulfed in innate immune cells, E: epithelium, Log: logarithmic scale, 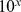: the number on the x-axis should be used as the exponent of 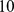 to obtain the true value.

The biological values of many of these quantities are unknown (Table 1). The goal of this study is to determine the degree of dependence of the predicted outcomes on the underlying parameters. We primarily focus on varying parameters about whose values we are most uncertain in the context of shigellosis. These are the antibody decay rates (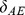, 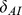, and 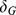), the rate antibodies neutralize *Shigella* (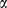 and 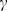), the B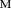 carrying capacities (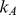 and 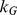), the rate B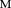 differentiate into plasma cells upon antigenic stimulation (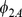 and 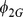), the number of plasma cells generated by proliferating antigen-activated B cells (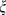), the rate that plasma cells are generated from antigen-activated B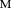 cells (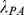, 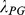, 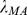, 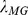), and the delay terms (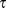 and the primary infection immune delay). Results of these simulations are shown in Figures 6, 7, 8.

Antibody half-lives have been measured clinically in humans [42]; yet, survival times for antibodies present in the lamina propria may be lower due to time spent localizing to the lamina propria, transcytosis, washout, and other factors. To better understand how antibody survivorship times affect immune and bacterial dynamics, we vary the antibody decay rates (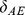, 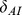, and 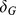), which we set to be equal to one another, from 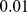/d to 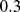/d. In Figure 6a, we plot the magnitude of the total post-vaccine antibody peak, which sums lamina propria IgG, lamina propria IgA, and luminal IgA. As the natural antibody death rate increases, the peak number of antibodies decreases because the time window over which antibodies present at the peak were created is broader. Figure 6b gives the time at which the antibody peak occurs as well as the time at which only 10% of the peak remains following the infection. Since we limit the time frame to 45 days, the total antibody population never decays to 10% of the peak for small antibody death rates. Comparing these figures, we see that smaller antibody peaks require less time to be reached. Despite the changing antibody peaks, the bacterial peaks do not vary much with the antibody death rates, as seen in Figure 6c, wherein we plot peak *Shigella* numbers in the lumen (L), lamina propria (LP), epithelium (E) or engulfed in innate immune cells (I). In Figure 6d, we plot *Shigella* peak times, which also are independent of this parameter. However, we also plot the time at which the total *Shigella* population in the lumen, lamina propria, and engulfed in innate cells drops below one bacterium, corresponding to *Shigella* extinction in non-epithelial compartments. This extinction time does increase with antibody death rates, likely because the amount of antibody capable of removing bacteria decreases.

We next vary the rate that antibodies neutralize *Shigella* in the lumen and lamina propria (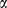 and 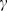) from 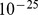 to 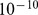/antibody/day. Our model value of 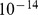/Ab/day was chosen for being the largest neutralization rate for which a non-epithelial *Shigella* infection takes at least a day to peak in simulations; it was not chosen as an optimal value that fits more biologically stringent *Shigella* peak behaviors. By varying the value, we see that higher neutralization rates correspond to faster, and thus lower magnitude, antibody peaks (Figures 6e–f). Only for smaller antibody peaks do antibody levels decay to 10% of the peak within 45 days. Little change in peak antibody numbers occurs for a neutralization rate lower than 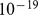/Ab/day, which suggests that this is a lower bound on antibody effectiveness. This is confirmed by examining the *Shigella* peak magnitudes (Figure 6g) and times (Figure 6h), which are identical for all neutralization rates below about 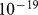/Ab/day. However, higher neutralization rates lower the peak bacterial load nonlinearly and drive non-epithelial *Shigella* infections to extinction within 0–3 weeks. The epithelial peak also never reaches above 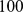 bacteria at day 45 for naturalization rates larger than 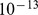/Ab/day (Figure 6g); however, such rates lead to the nearly immediate elimination of the *Shigella* infection (Figure 6h) and thus may not be biologically feasible. Whether increasing the neutralization ability of individual antibodies is possible and effective in clinical parameter ranges should be explored in more detail with future modeling.

Since the number of antigen-specific B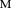 cells needed to confer immunity to *Shigella* is unknown and likely can vary with antigen targets, we vary the carrying capacities (i.e., the maximum sustainable cell numbers in the absence of infection) for IgA- and IgG-B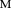 cells (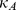 and 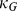) from 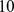 to 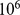 cells in Figures 6i–l. Increases in the carrying capacities induce roughly the same order-of-magnitude increase in the peak number of total antibodies but does not much affect the timing of the peak or the time at which all but 10% of the peak antibody remains (Figures 6i–j). Interestingly, the carrying capacity for *Shigella*-directed B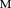 cells does substantially impact the amount of *Shigella* present in the epithelium (Figure 6k). If 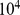 or more *Shigella*-specific IgA- and IgG-B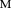 cells can be sustained, the peak number of *Shigella* in a post-vaccine infection at day 45 remains below 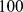 bacteria (Figure 6k). This is due in part to the resulting order-of-magnitude increase in both initial (disease-free equilibria) and peak antibody numbers when the carrying capacities are changed from 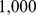 cells to 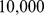 cells. However, Figure 4 suggests the presence of 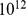 total antibodies prior to infection would still not be sufficient to confer protection; this figure assumes a pre-infection boost of antibodies above vaccine levels (via a serum injection of antibodies, for instance) without a corresponding increase in B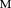 cells or other immunity. This makes clear that an antibody boost alone is not sufficient for immune protection. However, an antibody increase resulting from an underlying boost in B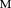 cells can confer protection if high enough B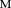 cells numbers are reached, perhaps because higher antibody levels can then be sustained for longer times. Importantly, from Figure 6k, we see that altering the B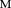 carrying capacity can improve the effectiveness of a vaccine enough that a purely anti-LPS *Shigella* vaccine could be sufficient to confer immunity. This suggests that anti-LPS B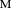 cells could serve as correlates of immunity and should be a key focus of future work in parallel with explorations of epithelial entry protein targeting.

To explore how antigen-stimulated plasma cell creation affects bacterial and immune dynamics, we look more closely at the rates that B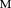-cells differentiate into new plasma cells in response to antigen (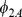 and 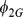) and at the number of plasma cells created per B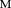 cell from this differentiation and subsequent proliferation (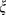). We vary the rates from 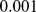 to 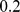 and see in Figures 7a–d that both the antibody and *Shigella* peaks are insensitive to large differentiation rates. When antigen-activated B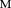 cells differentiate less frequently into plasma cells, the peak magnitudes for both *Shigella* and total antibody are higher, although the peak times vary only minutely. This increased antibody production despite lower ASC creation rates demonstrates that either (1) consistent with what we have seen previously, the minor increase in the time to the antibody peak is sufficient to create more antibodies, despite a lower plasma cell creation rate or (2) antigenic stimulation of B cell generation due to higher *Shigella* levels increases sufficiently to compensate for lower B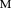 differentiation rates. The extinction time for non-epithelial *Shigella* drops only slightly when plasma cells are generated more quickly from B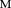 cells.

When we examine how the number of plasma cells made per differentiating B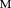 cell in the presence of antigen (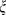) affects antibody peak dynamics, we see that antibody levels rise and *Shigella* numbers decay substantially when more plasma cells are made per B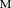 cell (Figures 7e–h). In these figures, the value of 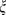 ranges from 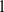 to 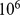. Here, higher antibody levels can be achieved with smaller peak times because ASC are more plentiful. Unlike with plasma cell rates, the extinction time for non-epithelial *Shigella* drops substantially when more plasma cells are generated per B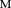 (Figure 7h); this suggests that it is the number, not the rate, of ASC production that matters for *Shigella* clearance. Furthermore, if more than 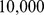 plasma cells are created by each antigen-stimulated B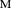 cell differentiation and proliferation, the day-45 bacterial load in the epithelium can be contain to less than 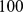 bacteria (Figure 7g). This assumes a B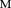 carrying capacity of 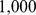 cells and indicates that if the B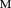 homeostatic number cannot be boosted, immune protection might be achievable if the plasma-cell-generating potential of each B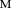 can be sufficiently increased.

To explore how the creation of new B cells from naive cells impacts infection dynamics, we first vary the creation rates of plasma cells from naive B cells (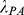, 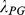, 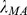, and 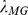) from nearly zero (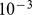) to 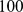 plasma cells/bacteria/day. After a vaccine, both *Shigella* and antibody dynamics are completely insensitive to changes of creation rates from naive B cells since pre-existing immunity contributions dominate (not shown). Thus, we also evaluate primary infection behavior. The faster that plasma cells are created from naive B cells, the higher the antibody peak and the more swiftly it is reached during a primary infection (Figures 8a–b). The higher antibody presence has little effect on the non-epithelial *Shigella* peaks, but the bacterial peak in the epithelium is reduced (Figure 8c). Furthermore, small plasma cell creation rates can hinder *Shigella* clearance during a primary infection, but rates above 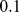 plasma cells/bacteria/day show little variation in *Shigella* extinction times (Figure 8d). Our arbitrarily chosen value of 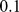 plasma cells/bacteria/day thus results in roughly the same dynamics as far higher plasma cell creation rates.

Lastly, we investigate how the time delays used in our model influence the results. We use two time delays: (1) the time delay (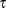) for new plasma cell and B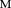 creation from naive B cells which serves as the delay component of the differential equations and (2) a numerically enforced initial delay before naive cell activation can occur during an infection. To evaluate the impact of these delays, we consider primary infection dynamics rather than post-vaccine dynamics, as the former is where variation will be most evident. Yet, the value of the delay component built into the model (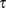), which we range from 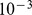 to 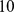 days, has little-to-no effect on any observed dynamics, as can be seen in Figures 8e–h. Theorizing that this might be due to the small value for the 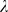
s, which multiply the delayed *Shigella* numbers, we set 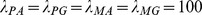 plasma cells/bacteria/day and reevaluated the delay effect. We again found no variation in the dynamics relative to 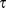. Hence, the use of delay differential equations at this stage was not essential to the observed results. However, we continue to use delay equations because it incorporates the biologically observed delay from naive B cell activation to effector or memory cell functionality without limiting our computational ability. Furthermore, it establishes a realistic modeling infrastracture that could be useful in future work.

The second delay, with which we allow the *Shigella* infection to establish for 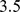 days before naive B cell activation, does impact the results. We implement this numerically by starting an infection at either a trivial or disease-free equilibrium but running the reduced system in which we eliminate the terms for the creation of B cells from naive cells (i.e., any terms with 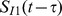 or 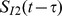 are set to zero) for 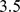 days. During this time, pre-existing immunity is unimpeded in its function or ability to generate new cells or antibodies. After 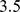 days, the naive cell terms are added back in and the full system runs from where the reduced system left off. When we vary this incubation time window from 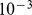 to 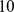 days, Figures 8i–l result. Time delays less than one day change the dynamics little relative to one another, but longer delays increase *Shigella* peak numbers, which results in higher antibody peaks due to increased antigenic stimulation. Clearance of *Shigella* varies only slightly even when the *Shigella* peak magnitude doubles. In fact, the *Shigella* extinction time without the incubation period is identical to the 20-day extinction time with a 3.5-day incubation period. Thus, quick naive B cell activation is not vital to clearance of a *Shigella* infection.

## Discussion

In this paper, we have established a mathematical framework for studying host immune interactions with *Shigella*, a dysentery-causing bacteria that kills over a million people worldwide every year. The ultimate goal of this work is to inform *Shigella* vaccine design by elucidating which immune components and bacterial targets are critical for establishing *Shigella* immunity, as “identification of the immunological correlates of protection is arguably the most crucial catalyst needed to accelerate development of effective *Shigella* vaccines” [3].

Our delay differential equation model focuses on humoral immune responses (antibodies and B cells) directed against specific antigens such as LPS in *Shigella*’s outer membrane. Bacteria are targeted by anti-LPS IgG antibodies and macrophages in the lamina propria and by anti-LPS IgA antibodies in the lumen. We examine both primary infection and post-vaccine/secondary infection dynamics by initializing simulations of our model at the trivial or disease-free equilibria, respectively. Since we do not incorporate first-wave, IgM antibodies nor CMI into our model, primary infection dynamics should be viewed as a best-case approximation to actual dynamics. Equilibrium analysis and numerical simulations reveal that anti-LPS antibodies clear the lamina propria and luminal infections but are unable to prevent epithelial invasion, which causes the most severe symptoms in the host. Thus, an antibody-based vaccine targeting only surface antigens cannot elicit sufficient immunity for protection.

To explore whether a larger amount of anti-LPS antibodies than a vaccine elicits can prevent disease, we boosted IgA and/or IgG antibody numbers prior to infection via initial conditions and tracked the number of *Shigella* bacteria in the epithelium 45 days later. A primary CMI response (not modeled) in concert with a post-vaccine humoral immune response should eliminate some small amount of epithelial cell invasion, although this threshold value is unknown. However, we find that a four-orders-of-magnitude boost in IgA and IgG numbers above vaccine levels is necessary to contain the epithelial infection below a threshold of 100 bacteria, a low estimate of the minimum infectious dose in humans [19], [39], [40]. Furthermore, this assumes a low proliferation rate of *Shigella* inside the epithelium and thus is likely an underestimate of antibody requirements. Therefore, it will be difficult to elicit sufficient numbers of antibodies targeting only *Shigella* LPS (or other outer membrane components) to prevent shigellosis.

The caveat is that if a *Shigella* vaccine can sufficiently boost not only anti-LPS antibodies but anti-LPS B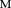 cells as well, the model predicts that protection might be achievable. Alternatively, if the ability of each B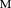 cell to generate plasma cells can be amplified, lower B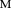 carrying capacities might be sufficient for protection. The importance of the B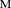 pool in this protection indicates that anti-LPS B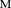 cells could be correlates of immunity for *Shigella*. However, more careful sensitivity analysis will be required to ensure that B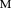 cells are correlates in experimentally relevant conditions. This is a promising research avenue for future mathematical studies.

While varying the initial IgA and/or IgG levels to determine the impact of antibody boosting, we are able to determine the relative effectiveness of IgA antibodies versus IgG antibodies. Although their efficacies are closely matched, the modeling results tell us that each IgA antibody is slightly more effective than an IgG antibody. Since the model’s IgA and IgG parameter values are identical, this likely results from the fact that IgA functions in the lumen and thus eliminates *Shigella* at the outset before it has the opportunity to proliferate. However, more total IgA is required than IgG to have an equal effect, because only IgA antibodies that reach the lumen contribute to this higher-per-molecule defense. A combined strategy with both IgA and IgG responses requires less total antibodies than IgA alone or IgG alone and thus is predicted to confer the best protection.

Since an anti-LPS antibody response is not sufficient for immunity, we extend the model non-mechanistically to explore the potential of additionally targeting *Shigella* epithelial entry proteins. We find that if IgG modulates the rate at which *Shigella* enters epithelial cells, epithelial invasion is almost entirely blocked. Thus, this vaccine strategy shows promise, yet a detailed, mechanistic model of epithelial entry that takes into account factors such as the brief availability of epithelial entry proteins is needed to better explore this avenue mathematically.

*Shigella* proteins involved in host cell invasion have long been considered potential vaccine targets. In fact, past vaccine trials have targeted both LPS and invasion plasmid antigens (IpaB, etc.), which play a key role in epithelial entry [43]. Thus, in future modeling, we will look more carefully at these elements to determine the mechanisms responsible for immune efficacy and to investigate other potential targets that alone or in combination can accurately predict vaccine effectiveness. Our current model thus serves as a launching point from which we can look more deeply at *Shigella* immune interactions in the future to better inform *Shigella* vaccine design.

Challenges exist in incorporating clinical data into the mathematical model. Human data are sparse in time and indirectly measured due to limited access to internal tissues, including the gastrointestinal mucosa. In addition, cut-offs for morbidity in patients are not quantitatively known, and thus it is not straight-forward to determine what bacterial loads and immune levels must be reached to achieve protection.

Our mathematical model includes many parameters that are biologically unknown (see Table 1). Our sensitivity studies are only an initial step in exploring the parameter space. While they provide insight into the role of individual parameters, much is left to be done in determining optimal clinical values and their resulting dynamics. An extensive investigation of the parameter space is beyond the scope of this work and is left to future studies and experimentation.

In summary, the mathematical model presented here explores several immune variables currently considered important in protection from *Shigella* infections. The model highlights the relative importance of the efficiency of IgA versus IgG, B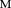 cells versus naive B cells, and optimized efficacy with elicitation of both IgA and IgG against LPS. It is expected that highlighting the importance of these variables, including additional ones such as CMI and other *Shigella* antigens, and continued testing of the model as additional clinical data become available will accelerate the development of vaccines against *Shigella.*
